# DNA methylation patterns in the peripheral blood of Xinjiang brown cattle with variable somatic cell counts

**DOI:** 10.3389/fgene.2024.1405478

**Published:** 2024-07-09

**Authors:** Dan Wang, Shengchao Ma, Mengjie Yan, Mingming Dong, Menghua Zhang, Tao Zhang, Tao Zhang, Xiaoxue Zhang, Lei Xu, Xixia Huang

**Affiliations:** College of Animal Science, Xinjiang Agricultural University, Urumqi, China

**Keywords:** DNA methylation, mastitis, somatic cell count, Xinjiang brown cattle, *CD4* and *TRAPPC9* gene

## Abstract

The use of wide-ranging dairy herd improvement (DHI) measurements has resulted in the investigation of somatic cell count (SCC) and the identification of many genes associated with mastitis resistance. In this study, blood samples of Xinjiang brown cattle with different SCCs were collected, and genome-wide DNA methylation was analyzed by MeDIP-seq. The results showed that peaks were mostly in intergenic regions, followed by introns, exons, and promoters. A total of 1,934 differentially expressed genes (DEGs) associated with mastitis resistance in Xinjiang brown cattle were identified. The enrichment of differentially methylated CpG islands of the *TRAPPC9* and *CD4* genes was analyzed by bisulfate genome sequencing. The methylation rate of differentially methylated CpGs was higher in the *TRAPPC9* gene of cattle with clinical mastitis (mastitis group) compared with healthy cattle (control group), while methylation of differentially methylated CpGs was significantly lower in *CD4* of the mastitis group compared with the control group. RT-qRCR analysis showed that the mastitis group had significantly reduced expression of *CD4* and *TRAPPC9* genes compared to the control group (*p* < 0.05). Furthermore, Mac-T cells treated with lipopolysaccharide and lipoteichoic acid showed significant downregulation of the *TRAPPC9* gene in the mastitis group compared with the control group. The identified epigenetic biomarkers provide theoretical reference for treating cow mastitis, breeding management, and the genetic improvement of mastitis resistance in Xinjiang brown cattle.

## 1 Introduction

Bovine mastitis, an inflammation of the mammary gland, represents an imbalance between the susceptible animal, the pathogen, mechanical causes, and the environmental factor (Cheng and Han, 2020). Inflammation is the most common pathophysiological response to bacterial invasion and proliferation in animals and can be induced by chemical factors, thermal factors, or mechanical damage (Ruegg, 2017). There are many causes of bovine mastitis, associated with a variety of factors. Some pathogenic bacteria create toxins that directly injure the mammary gland, reducing milk production and most likely resulting in the cow’s death, shortening the dairy cattle replacement cycle (Bar et al., 2007; Cheng and Han, 2020). The somatic cell count (SCC) is an indicator of raw milk quality in dairy cattle and has a strong genetic correlation with mastitis (Sender et al., 2013). The SCC can not only predict the likelihood of mastitis in dairy cattle but also reflect the quality of the milk. Therefore, the SCC is currently the trait of choice for improving mastitis resistance (Miglior et al., 2009). Although antibiotics and antimicrobials are important in the treatment of mastitis in cattle, breeding for disease resistance is the most efficient and long-term approach to fighting pathogen invasion.

Changes in the gene sequences and the genome structure may alter the phenotype of animals and can be stably passed on to their offspring (Jones, 2012). In the study of genome structure, the concept of epigenetics was proposed by Waddington in 1939. The surfaces of nucleosomes are studded with a multiplicity of modifications. At least eight different classes have been characterized to date, and many different sites have been identified for each class (Kouzarides, 2007). Currently, the main areas of interest in epigenetics are DNA methylation, histone modification, and non-coding RNAs. DNA methylation is usually analyzed using genome-wide and site-specific approaches. The genome-wide analysis involves a variety of techniques, such as MeDIP-seq, WGBS, HPLC, and others. The methods used for site-specific DNA methylation analysis include BSP, COBRA, MSP, and other methods (Li et al., 2012; Cortijo et al., 2014). DNA methylation is involved in the regulation of numerous biological processes, including growth and development, disease resistance, and reproduction, as well as other livestock traits to varying degrees (Jones and Takai, 2001). For instance, Dechow et al. identified 72 differentially methylated regions (DMRs) between high milk-yield cows and controls (Dechow and Liu, 2018). In mastitis, a common and complex disease affected by genetics, environment, and pathogens with a high incidence rate, several effective epigenetic markers have been identified. Wang et al. constructed a genome-wide DNA methylation map of *Staphylococcus* aureus-induced mastitis in Chinese Holstein cattle and found that C^m^CGG differentially methylated/expressed genes, such as *IL6R*, *TNF*, *BTK*, *IL1R2*, and *TNFSF8*, were enriched in several immune-related Gene Ontology (GO) terms, indicating their important roles in the host immune response and their potential as candidate genes for mastitis caused by *S. aureus* (Wang et al., 2020). These results, together with the findings of previous studies on genes associated with mastitis resistance, were reviewed, and the *TRAPPC9* and *CD4* genes were selected for in-depth analysis.

Trafficking protein particle complex 9 (*TRAPPC9*) is located on cattle chromosome 14 (GENE ID: 533451) and encodes a NIK- and IKK-b-binding protein (NIBP). Its role in intellectual disability has been thoroughly investigated. Mir et al. identified a truncating homozygous mutation, R475X, in exon 7 of the *TRAPPC9* gene that was associated with nonsyndromic autosomal-recessive intellectual disability (Mir et al., 2009). Increases in TRAPPC9 boost methicillin NF-κB signaling during mastitis in dairy cattle (Khan et al., 2022). Song et al. (2016) investigated methicillin-resistant *Staphylococcus aureus* infection of mammary epithelial cell lines and found substantial downregulation of *TRAPPC9* expression 6 h after infection (*p* < 0.05). The cluster of differentiation 4 (*CD4*) gene is located on chromosome 5 in cattle (GENE ID: 407098). *CD4* is an accessory protein that binds non-covalently to the T cell receptor and recognizes an invariant region of MHC class II proteins on antigen-presenting cells (Napolitano et al., 2021).

Cattle mastitis is caused by pathogens such as *S. aureus*, *Escherichia coli*, and *Streptococcus*. Infection of the mammary gland during lactation leads to clinical mastitis, which causes a rise in SCC in milk and CD4^+^ T lymphocytes. Wang et al. (2013) observed that Chinese Holsteins with clinical mastitis (CM) have 16% more methyl groups (75.0% ± 5.8%) in their *CD4* promoter than healthy controls (59.0% ± 8.5%) (Wang et al., 2013). The DNA methylation status of CpG islands in the *CD4* promoter region in cattle with CM (the mastitis group) significantly affected the expression of *CD4* in the blood. Additionally, the analysis of associations between the selected haplotypes in the *CD4* gene and milk related indexes showed that bulls with Hap2 (T-A-C-C) had improved indexes for milk and protein yields (Napolitano et al., 2021). The frequency of methylation at CpG_2 was found to be significantly correlated with *CD4* mRNA expression in Dapulian (DP)/Landrace hybrid pigs (Zhao et al., 2018). In the present study, *TRAPPC9* and *CD4* were used as candidate genes for mastitis resistance, and the BSP method was used for analysis of methylation at the single-base level to identify molecular markers related to mastitis resistance and susceptibility in Xinjiang brown cattle.

Xinjiang brown cattle have been widely acknowledged by farmers and herdsmen for their cold resistance, tolerance to roughage in feed, and high resistance to stress. Recently, the specific characteristics of Xinjiang brown cattle have received much attention. Wang et al. compared Chinese Holstein cattle with Xinjiang brown cattle under the same feeding management conditions and found that Xinjiang brown cattle had the advantage of low SCC (Wang et al., 2014). Furthermore, genes (e.g., the *FHIT* gene) associated with the somatic cell score (SCS) in Xinjiang brown cattle were identified by a genome-wide association study (GWAS) (Zhou et al., 2019). Zhong et al. revealed that the promoter methylation levels of the *FHIT* and *PIAS1* genes in the mastitis group were higher and lower, respectively, than those in the healthy group (Zhong et al., 2023) and Ju et al. reported that 8 of 13 InDel loci were polymorphic in the *FHIT* gene, with all the new InDel variants significantly related to six different milk traits (*p* < 0.05) (Ju et al., 2021). However, mastitis resistance in cattle involves a complex genetic architecture. For further investigation of the unique genetic resource represented by Xinjiang brown cattle, the present study aimed to document the landscape of DNA methylome distribution in the bovine peripheral lymphocyte genome of healthy cattle and cattle with CM, as well as to analyze two novel DNA methylation target genes (*TRAPPC9* and *CD4*) that were correlated with mastitis in Xinjiang brown cattle.

## 2 Materials and methods

### 2.1 Sample collection

A total of six Xinjiang brown cattle were selected from Xinjiang Yanben Brown Cattle Breeding Development Co., Ltd. The DHI records of this farm were monitored continually, and healthy cattle and cattle with CM with the same feeding management conditions and the same lactation period with SCC ≤200,000 cells/mL or SCC ≥5,000,000 cells/mL in the DHI data for 6 consecutive months were selected and assigned to different groups (low SCC groups: L6, L8, L0; high SCC groups: H3, H9, H12). 5 mL of blood were collected from the tail veins into EDTA-K2 anticoagulation tubes and stored at −40°C for DNA extraction. Finally, 10 mL of blood was further collected in EDTA-K2 anticoagulant tubes and centrifuged at 3,000 rpm for 15 min to collect the white membrane layer for RNA extraction; 50 mL of milk was collected for measurement of SCC.

### 2.2 DNA and RNA extraction

DNA was extracted from the blood DNA using the phenol-chloroform method. The genomic DNA was electrophoresed on 0.8% agarose gels containing nucleic acid dye using fragments labeled by λ-Hind III digest as a marker in 0.5×TBE buffer, 110 V, for 25 min. The DNA was quantified, and its quality was assessed by NanoDrop 2000. Total RNA was extracted from the blood samples using the PAXgene Blood RNA Kit.

The purity and concentration of the RNA were assessed using agarose gel electrophoresis and an Agilent 2100 bioanalyzer (Santa Clara, CA, United States). Total RNA was extracted from MAC-T using TRIzol reagent (Invitrogen, Carlsbad, CA, United States).

### 2.3 MeDIP-seq

The quality-assured DNA was fragmented and end-repaired, and an A was added to the 3′end to avoid the formation of chimeras by interlinking of the DNA fragments. The junction was ligated, and the ligated purified product (about 400 ng) was incubated with a DNA methylation-specific antibody for immunoprecipitation (IP) at 37°C for 0.5–1 h. The product was then washed, collected, amplified, and purified, and quality control was performed. Sequencing was performed on an Illumina-HiSeq 2500 platform (Illumina, San Diego, CA, United States). After sequencing, the quality of the pre-processed sequencing data from six Xinjiang brown cattle samples was assessed, the raw image files were filtered by base identification and error filtering to obtain reads for analysis, and the results were stored in FASTQ format. The results included information on the base composition of the sequences and their corresponding sequence quality. The evaluation criteria were based on the sequencing quality’s Q-values. The relationship between the Q-value and sequencing error E-value was Q = −10Log10E, and the Q-value box plot statistic and the base distribution graph were used to evaluate the raw data.

### 2.4 Analysis of peak inter-sample differences

The clean reads from the sequencing were used for data analysis, followed by filtering with Fastx (version 0.0.13) (HannonLab, 2014). Genome mapping was performed with Bowtie (version 0.12.8) (Langmead et al., 2009), and the clean read sequences were aligned with the reference genome (Bos_taurus_UMD_3.1.1) to obtain bam files. MACS (version 1.4.2) (Feng et al., 2012) was used to locate the peak-enriched regions of the bam files. Pie charts and histograms were plotted through statistical analysis.

Using the information on the positions of the peaks, the peaks and genes were annotated, together with the functional elements of the genes (promoter, 5′UTR, coding regions, 3′UTR, introns, and TTR, where the promoter was 2000 bp upstream of the transcription start site and the TTR was 5000 bp downstream of the transcription termination site) and CpG islands (where the shores were 2000 bp upstream and downstream of the CpG islands, and the shelves were 2000–4000 bp upstream and downstream of the CpG islands).

### 2.5 DMR analysis and annotation

The differences between the peaks of the healthy cattle and the cattle with CM were analyzed using the R package MEDIPS. The default parameter setting was |Fold change| ≥ 2, and *p* < 0.05, and the resulting DMR was then annotated. The annotation program was the same as that used for the peak annotation, using parameters such as differential peak position and annotation information. The DMR obtained from the analysis was used for chromosome region distribution. The DMR-related gene information in each term in the GO database (https://geneontology.org/) was recorded for each entry. GO terms and KEGG pathways showing significant enrichment in DMR-related genes were used in DAVID (Sherman et al., 2022). The PPI network was mapped using the STRING version 12.0 database and visualized using Cytoscape version 3.9.0.

### 2.6 BSP

BSP was used to verify the level of DNA methylation in the DMR regions of key candidate genes. Eight Xinjiang Brown cattle were selected; these included all the sequenced individuals. Blood was collected, and DNA was extracted. The bisulfite modification of DNA was performed using an EZ DNA Methylation-Gold Kit (Zymo Research, Los Angeles, United States), and significant DMRs were selected according to the sequencing results. Primers were designed and amplified using the enrichment of reads at different positions of the genome obtained from the comparison results. The recovered PCR products were ligated with the PMD 19-T vector, and the algorithm of the PCR product mole number was as follows: the amount of DNA ready to be ligated (ng) = nmol×660×bp number of the DNA ready to be ligated. The ligation products were transformed into DH5α competent cells, coated on LB Amp^+^ plates, and incubated at 37°C overnight. Single colonies were picked, inoculated into LB liquid medium, and cultured overnight for PCR identification; positive clones were picked from the coated plates and subjected to Sanger sequencing. The primers and BSP amplification system are shown in Supplementary Table S1.

### 2.7 MAC-T cell culture and induction of inflammation

Mac-T cells were cultured in high-glucose medium (DMEM, Biological Industries, Israel) supplemented with 10% fetal bovine serum (FBS, VivaCell, China) and 2% penicillin-streptomycin (Solarbio, China). The cell culture incubator was set at 37°C with 5% CO_2_. The negative control group (NC) was set up in 12-well cell culture plates, while the LPS group was treated with 5 μL (10 ng/μL) of LPS and cultured for 3 h, and the LTA group was treated with 20 μL (20 ng/μL) of LTA and cultured for 12 h, respectively, and the cells were collected with three replicates for each treatment.

### 2.8 RT-qPCR

After evaluating the quality and concentration of the RNA, it was reverse-transcribed into cDNA using the AG (AG11711) reverse-transcription kit. qPCR was performed using a 2 × Taq SYBRGreen^®^ qPCR Mix (Innovagene, China) using the primers shown in Supplementary Table S2. The reaction system (10 μL) included 5 ng of cDNA, 0.5 μL of each upstream and downstream primer, 5 μL of the SYBRGreen Mix, and 3 μL of ddH_2_O. The reaction procedure was as follows: 95°C for 3 min, followed by 40 cycles of 95°C for 5 s, 60°C for 20 s, and extension at 72°C for 5 s and 40 cycles. Three replicates were determined for each sample. *GAPDH* was used as the reference gene, and the relative expression of *TRAPPC9* and *CD4* genes was calculated using the 2^−ΔΔCt^ method.

**FIGURE 1:**
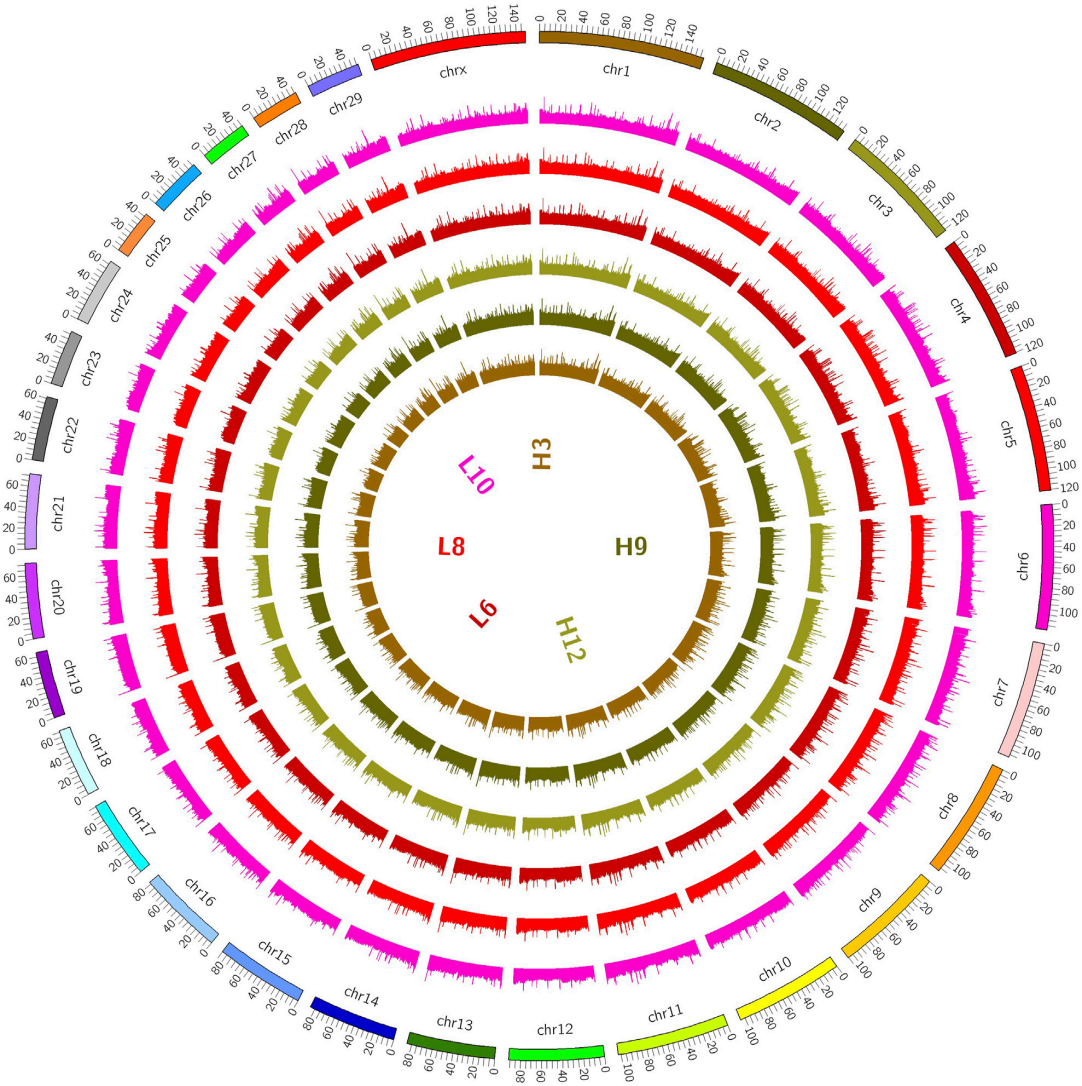
Distribution on chromosomes. (The coverage of the genome is shown in a 100K window, and the methylation level of the genome is indicated. The outermost circle in the figure represents the genome, and the methylation level of the sample genome is indicated in the inner circle).

### 2.9 Data analysis

The experimental data were preprocessed using GraphPad Prism 8.0.1, and independent sample t tests were conducted with SPSS 19.0. All values are shown as the mean ± SE, with a *p*-value of 0.05 as the threshold.

## 3 Results

### 3.1 MeDIP-Seq data processing and quality assessment

The extracted DNA samples showed clear bands, low degradation, and no contamination (Supplementary Figure S1). The sequencing reads were evaluated for overall quality, and the raw data were evaluated using Q-box plot statistics and base distribution plots (Supplementary Figure S2A). The results showed that the average Q-values of the six samples were all in the green background part (indicating high quality), suggesting that the sequencing Q-values met the quality criterion of Q20 values above 97% (Supplementary Table S3). The GC and AT base pairs were evenly distributed (Supplementary Figure S2B), and the quality of 90% of the reads was above 39 points (Supplementary Figure S2C). After conducting MeDIP-seq on the genomic DNA of Xinjiang brown cattle with different SCCs, the raw data obtained from sequencing were filtered to obtain 270,998, 164 high-quality sequence reads (clean reads), accounting for 95.98%–96.98% of the raw reads. The clean ratio of the six samples was above 95%, and the Q 20 value of all samples was above 95%, indicating that the individual base error rate was very low, indicating the reliability of the read quality produced in the preliminary database construction process (Supplementary Table S4).

### 3.2 Genome distribution trends

**FIGURE 2:**
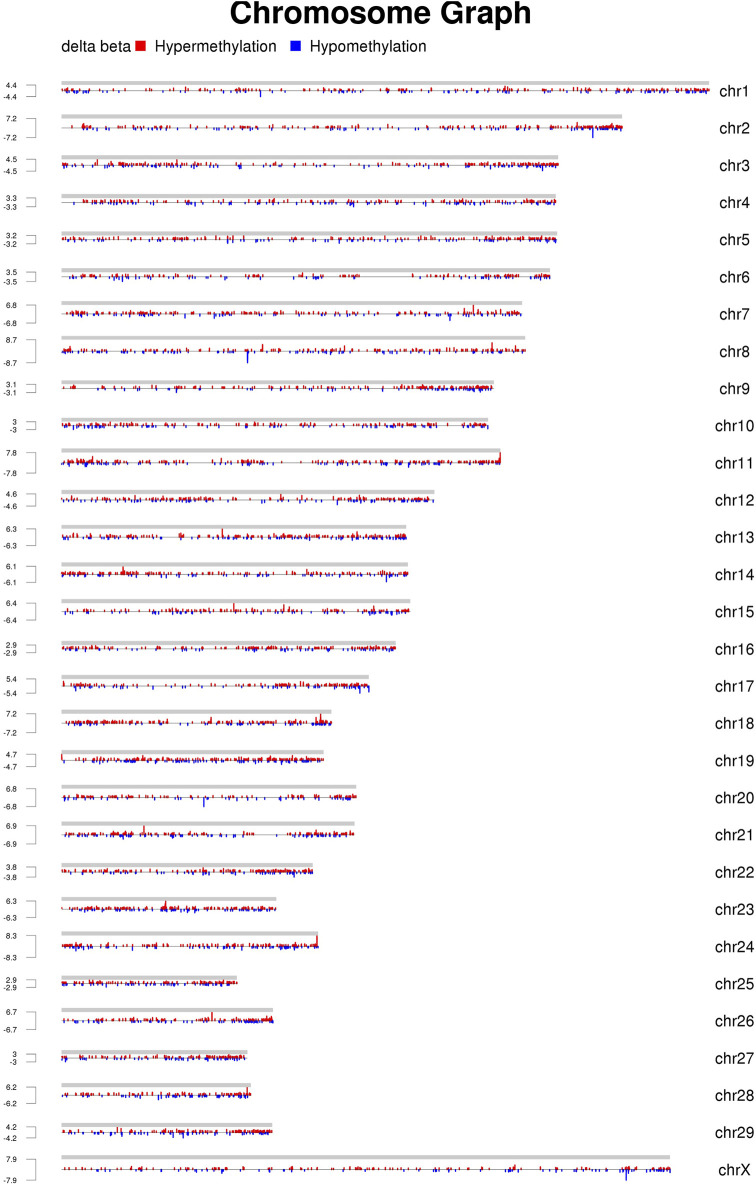
Distribution of MeDIP-seq reads on each chromosome (the chromosomal distribution of DMRs obtained using MEDIPS are shown. The differences in each group are shown on a single graph, with red indicating a high methylation level, blue indicating a low methylation level, and height indicating logFC, i.e., the foldchange).

The distributions of the reads on the genes and chromosomes were analyzed by comparing genomes. The analysis of genome coverage in each sample showed even distribution over the different chromosomes, with no obvious preferences (Supplementry Figure S12D). The length of the highly methylated fragment peak was about 500–1000 bp; enrichment of reads decreased gradually as the fragment length increased (Supplementary Figure S2E); and the reads of the two groups of samples were more enriched in the tail ends of each chromosome than in the other regions (Figure 2).

A total of 69,718 methylation peaks were identified in cows in group H3 of Xinjiang brown cattle with high SCCs, while 57,734 methylation peaks were observed in those in the L6 group with low SCCs (Supplementary Figure S3A). The peaks were found mostly in the intergenic regions, followed by introns, exons, and promoters. The genome of Xinjiang brown cattle contained a large number of CpG islands (CGIs). Statistical analysis of CGIs revealed that only 4.20–4.58% of the peaks were located in the CpG islands (Supplementary Figure S3B).

### 3.3 DMR cluster analysis

**FIGURE 3:**
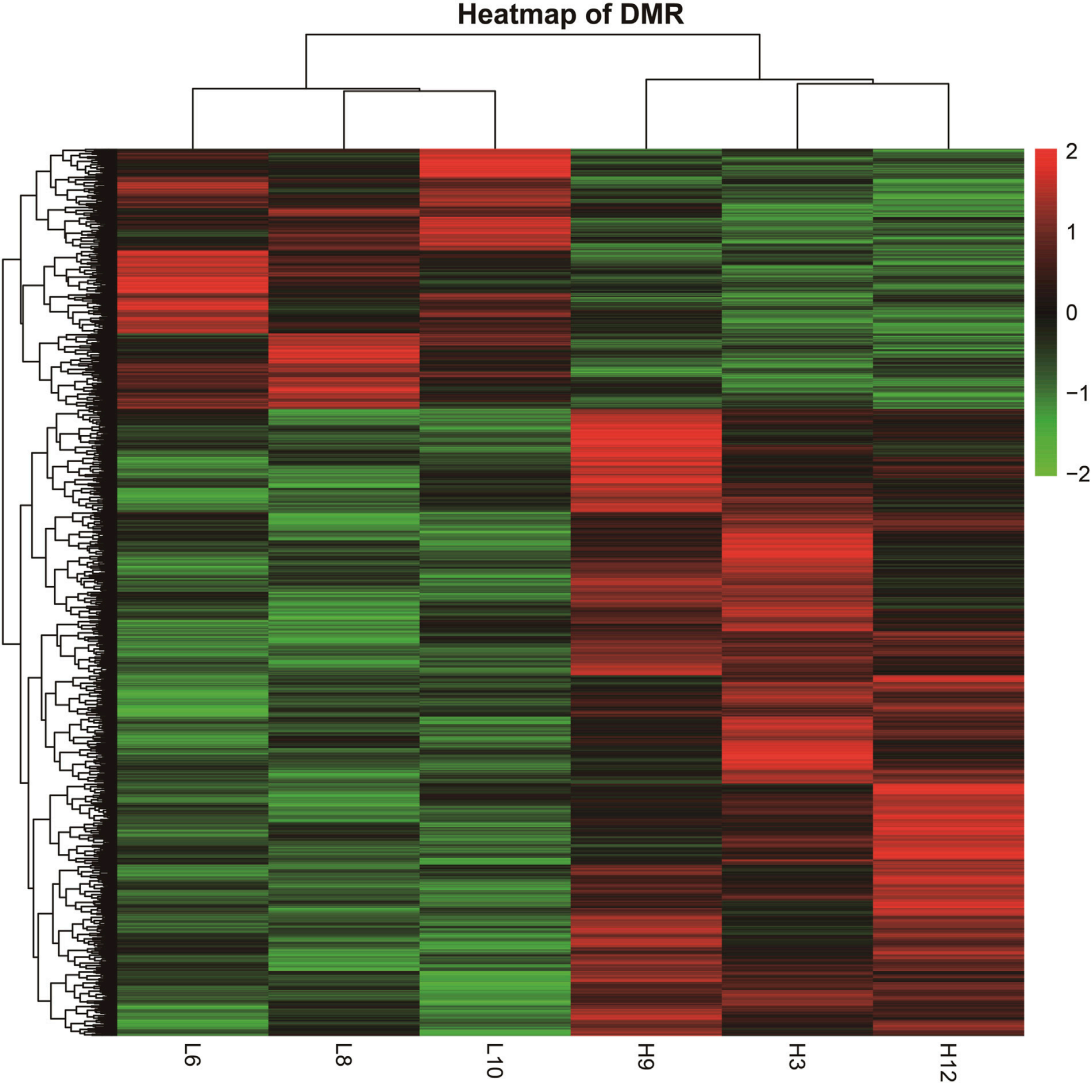
Heatmap of DMRs from different samples. (The heatmap was derived by RPKM of the DMRs of six samples. The samples are clustered horizontally and DMR is clustered vertically; red color indicates upregulation and green color indicates downregulation).

The DMRs shared by the six samples were selected, and the reads per kilobase per million mapped reads (RPKM) values were used to generate a heatmap for DMRs (Figure 3). The overall DMRs varied among cows with different SCCs and differed in any two modes, and the overall expression mode of DMRs in the high-SCC groups of the two experimental subgroups was very different from that in the low-SCC group of the control group: in the control group, most of the regions were downregulated (green bands), while the experimental group was exactly the opposite, with most of the regions showing upregulation. The expression trends were similar within each group. The reproducibility of the samples in each group was relatively high.

### 3.4 GO functional enrichment and pathway analysis of differently methylated genes (DMGs)

**FIGURE 4:**
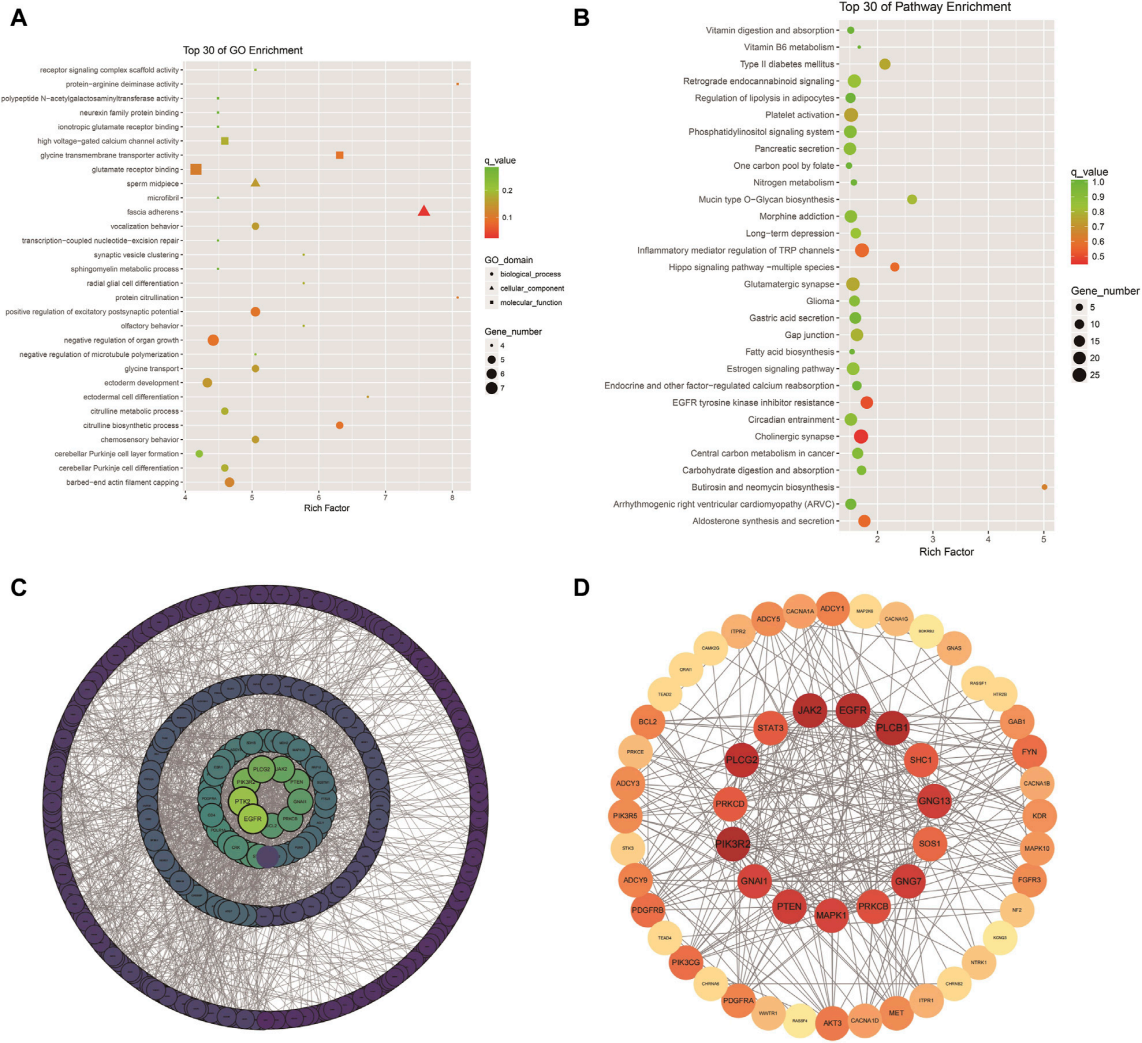
GO and KEGG enrichment: Scatter plot of the top 30 GO enrichments **(A)**; Scatter plot of the top 30 KEGG enrichments; **(B)** PPI network of DEGs; **(C,D)** PPI network of key genes in Table 1.

The analysis of Xinjiang brown cattle with different SCCs revealed that 1,717 genes were enriched in the GO biological process category, accounting for 73.87% of the total. A further 246 genes were enriched in the molecular function category, accounting for 10.57% of the total, while 362 genes were enriched in the cellular component category, accounting for 15.56% of the total. The significantly enriched GO terms were mainly associated with protein binding, cellular processes, biological processes, multicellular biological processes, cellular stimulus response, stress response, metabolic processes, redox reactions, and fascial adhesion. Among them, 27 genes were enriched in glutamate receptor binding, receptor signaling complex scaffold activity, and methylation, suggesting that these played important roles in biological processes, including 22 upregulated and 5 downregulated genes (Figure 4A). To explore the signaling pathways in which the DMGs were involved, KEGG analysis was performed, showing that 289 KEGG pathways were enriched (Figure 4B). These were involved in inflammation and cancer occurrence and development and included cholinergic synapses, EGFR tyrosine kinase inhibiting drug resistance, the hippo signaling pathway, inflammation regulation of the TRP channel, and aldosterone synthesis and secretion, all of which were related to processes linked with immunity, disease, and the bacterial invasion of epithelial cells (Table 1). The DEGs in these pathways may play an important role in the occurrence of cattle mastitis. The protein-protein interaction network (PPI) networks of DMGs (minimum required interaction score, 0.7) and key pathway-enriched genes (minimum required interaction score, 0.4) were mapped using the STRING V12.0 database. As indicated, genes such as *PIK3R2*, *PLCB1*, *EGFR*, *JAK2*, *PLCG2*, *GNG7*, *GNG13*, *PTEN*, *GNAI1*, *MAPK1*, *PRKCB*, *PRKCD*, and *STAT3* genes were located at the core of the network, indicating that these genes were related to mastitis in Xinjiang brown cattle (Figures 4C, D).

**TABLE 1:** Key pathways and genes for enrichment of DMR-related genes.

Pathway ID	Pathway	Gene number	Gene list (UP)	Gene list (DOWN)	*p*-Value
bta04392	Hippo signaling pathway -multiple species	9	TEAD2, RASSF1, LOC520730, TEAD4, LIMD1	STK3, WWTR1, RASSF4, NF2	0.014
bta01521	EGFR tyrosine kinase inhibitor resistance	20	FGFR3, JAK2, MET, PIK3CG, SHC1, SOS1, PLCG2, PDGFRA, BCL2, AKT3, PTEN, PIK3R2, PIK3R5, GAB1, EGFR, PRKCB, KDR	NRG, STAT3, PDGFRB	0.011
bta04925	Aldosterone synthesis and secretion	19	ADCY1, ORAI1, PLCB1, PRKCE, ITPR1, ITPR2, KCNK9, CAMK2G, ADCY3, CACNA1D, ADCY5, GNAS, CACNA1G, ADCY9, SCARB1, DAGLB, PLCB3, ATF1, PRKCB	-	0.016
bta04750	Inflammatory mediator regulation of TRP channels	26	BDKRB2, ADCY1, PLCB1, ACCN5, PRKCE, ITPR1, ITPR2, ACCN1, PIK3CG, CAMK2G, PRKCH, ADCY3, PLCG2, ADCY5, GNAS, HTR2B, PRKCD, ADCY9, MAPK1, PIK3R2, PIK3R5, PLCB3, NTRK1, MAP2K6, PRKCB	MAPK10	0.008
bta04725	Cholinergic synapse	27	CACNA1A, ADCY1, PLCB1, JAK2, ITPR1, CHRNB2, ITPR2, PIK3CG, CAMK2G, ADCY3, CACNA1D, ADCY5, CACNA1B, ADCY9, BCL2, AKT3, PIK3R2, KCNQ3, GNG13, PIK3R5, PLCB3, FYN, KCNQ, PRKCB	GNG7, CHRNA6, GNAI1	0.008

### 3.5 Methylation modifications of *TRAPPC9* and *CD4* genes in Xinjiang brown cattle with different SCCs

The findings of previous studies and the present analysis indicated that the expression of *TRAPPC9* and *CD4* genes was regulated by methylation. Therefore, these genes were considered candidate genes for further investigation into mastitis resistance in Xinjiang brown cattle.

**FIGURE 5:**
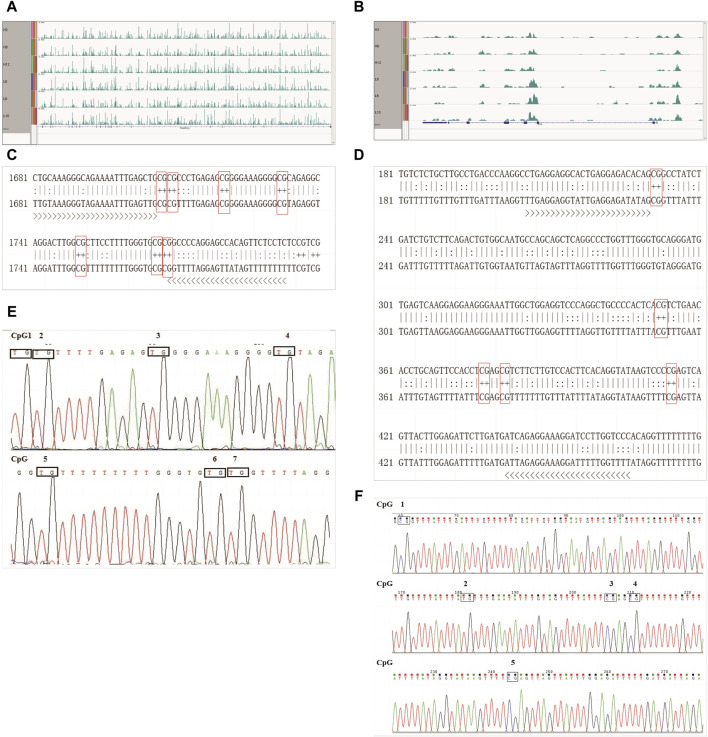
CpG sites of *TRAPPC9* and *CD4* genes: **(A)** Enrichment of *TRAPPC9* reads; **(B)** Enrichment of *CD4* reads; **(C)** Methylated CpG sites in *TRAPPC9*; **(D)** Methylated CpG sites in *CD4*; **(E)** Sequencing peak map of methylation regions of *TRAPPC9* gene; **(F)** Sequencing peak map of methylation regions of *CD4* gene.

First, the methylation modifications of the full-length *TRAPPC9* and *CD4* genes were investigated (Figures 5A,B). MethPrimer online software was used to analyze the location of the CpG islands in *TRAPPC9* gene, and DNA methylation primer sequences were designed for this region. The target fragment was 1681–1793 bp (Supplementary Figure S4A), and the amplification product size was 113 bp, which contained 7 potential DNA methylation sites (Supplementary Figure S4B). The target fragment of the DMR for *CD4* gene was 206–466 bp (Figure 5C), with a product size of 268 bp, containing five potential DNA methylation sites (Figure 5D). After confirmation of the genome DNA and PCR products (Supplementary Figure S5), DNA methylation of the *TRAPPC9* portion and the DMR target fragment of *CD4* was analyzed. The methylation pattern of Xinjiang brown cattle with different SCCs was analyzed using the temperature denaturation method based on the principle of cytosine conversion to uracil between cytosine and sodium bisulfite using the methylation kit. Figure 5E shows the sequencing peaks of the *TRAPPC9* methylation region of Xinjiang brown cattle, wherein seven methylation sites are present. Figure 5F shows the sequencing peaks of the *CD4* methylation region of Xinjiang brown cattle, wherein five methylation sites are present.

**FIGURE 6:**
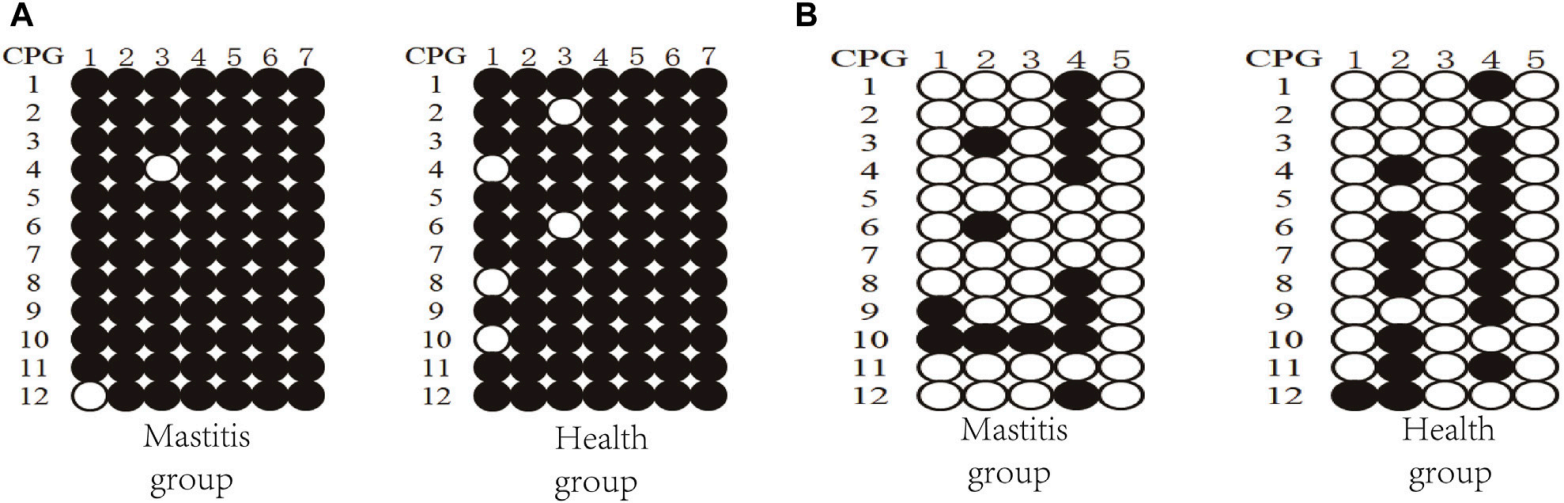
DNA methylation patterns of Xinjiang brown cattle with different SCCs: CpG sites in **(A)** DMR of *TRAPPC9* gene and **(B)** DMR of *CD4* gene.

Three clones from four Xinjiang brown cattle with CM and four Xinjiang brown cattle with normal lactation were analyzed, and the results of *TRAPPC9* bisulfite treatment and sequencing are shown in Figure 6A. Taking the methylation level of healthy cattle as a reference, the methylation rates of CpG2, CpG4, CpG5, CpG6, and CpG7 were higher in the mastitis group as well as in the control group. Meanwhile, the methylation rate of CpG in the mastitis group (97.62%) was higher than that in the control group (94.05%), while the methylation rate of CpG1 in the mastitis group (91.67%) was higher than that in the control group (75%), and the methylation rate of CpG3 in the mastitis group (91.67%) was higher than that in the control group (83.33%).

The results of the *CD4* bisulfite treatment and sequencing of Xinjiang brown cattle are shown in Figure 6B. Using the methylation level of healthy cattle as a reference, the methylation rates of CpG2 and CpG4 were found to be higher in both the mastitis and control groups. The methylation rate of CpG islands in the mastitis group (23.33%) was lower than that in the control group (28.33%), while the methylation rate of CpG1 in the mastitis group (16.67%) was higher than that in the control group (8.33%), the methylation rate of CpG2 was higher in the control group (58.33%) than that in the mastitis group (25%), the methylation rate of CpG3 in the mastitis group (8.33%) was higher than that in the control group (0%), and the methylation rate of CpG4 in the control group (75%) was higher than that in the mastitis group (66.67%).

### 3.6 Analysis of *TRAPPC9* and *CD4* expression in Xinjiang brown cattle

**FIGURE 7:**
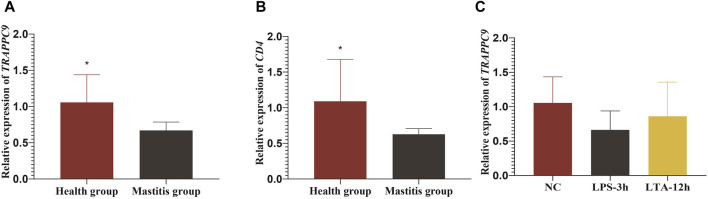
Relative expression levels of *TRAPPC9* and *CD4* genes. **(A)** and **(B)** Relative expression level of *TRAPPC9* and *CD4* genes in healthy cattle and cattle with mastitis. **(C)** Relative expression levels of TRAPPC9 in MAC-T cells.

The RT-qPCR results showed that both the *TRAPPC9* and *CD4* genes were expressed in the blood of cattle in the healthy and mastitis groups, and the expression levels of both genes were significantly higher in cows from the control group than those from the mastitis group (*p* < 0.05) (Figures 7A,B).

To elucidate the molecular mechanism underlying *TRAPPC9* involvement in the inflammatory response in mammary epithelial cells, the expression of *TRAPPC9* was assessed in MAC-T cells after treatment with 10 ng/μL LPS for 3 h and 10 ng/μL LTA for 12 h, respectively. It was found that both the LPS- and the LTA-treated groups showed reduced expression of TRAPPC9 compared with the control group (Figure 7C).

## 4 Discussion

Genetic tests are widely used for disease diagnosis, and DNA methylation is now recognized as an important component of precision medicine. Genome-wide association studies have revolutionized the study of complex human traits by identifying thousands of genetic loci that contribute to susceptibility to a diverse set of diseases, although the underlying nucleotide changes and mechanisms remain largely unknown (Farh et al., 2015). Gene expression in cells is modulated by epigenetic modifications of chromatin. Therefore, the study of epigenetic changes is very important to understand gene regulation at the molecular, cellular, tissue, and organ levels. DNA methylation is one of the most studied epigenetic modifications and has been shown to play an important role in maintaining the stability of the genome and ensuring normal growth and development (Zhang et al., 2023). For instance, the dynamic processes and precise control of molecular mechanisms during the maintenance of DNA methylation provide an important molecular basis for explaining abnormal changes in DNA methylation that occur during aging or in tumors (Ming et al., 2020). For instance, abnormal hypomethylation of *STAT3* has been identified as a biomarker for chronic benzene poisoning (Yang et al., 2014). The analysis of DNA methylation patterns is increasingly reliant on sequencing-based analytical methods. Harris et al. applied MethylC-seq, reduced representation bisulfate sequencing (RRBS), MeDIP-seq, and methyl-binding domain sequencing (MBD-seq) to human embryonic stem cells to assess their genome-wide CpG coverage, resolution, cost, concordance, and the influence of CpG density and genomic context, and found that the combination of MeDIP-seq and methyl-sensitive restriction enzyme sequencing (MRE-seq) was able to provide comprehensive coverage of methylation at a lower cost (Harris et al., 2010). Individual cows can experience changes due to a range of reasons, including some pathogenic bacteria releasing toxins that directly impact the mammary glands, potentially leading to milk loss and shorter life spans in severe cases. The development of molecular approaches provides guidance for identifying biomarkers for mastitis and developing novel molecular diagnostic procedures.

The methylation patterns in DNA derived from the blood of Xinjiang brown cattle with different SCCs were analyzed by MeDIP-seq. A total of 1,934 unique DEGs corresponding to DNA DMRs were identified, and these genes were found to be enriched in pathways related to metabolism, cellular processes, biological systems, and disease immunity, including cholinergic synapses, EGFR tyrosine kinase inhibiting drug resistance, the hippo signaling pathway, and inflammation regulation of the TRP channel. Further analysis of DMR-associated genes identified a variety of genes closely linked to mastitis in cattle, namely, *PIK3R2*, *PLCB1*, *EGFR*, *JAK2*, *PLCG2*, *GNG7*, *GNG13*, *PTEN*, *GNAI1*, *MAPK1*, *PRKCB*, *PRKCD*, and *STAT3*, among others, suggesting the potential involvement of these genes in regulating the occurrence and development of mastitis in Xinjiang brown cattle. The EGFR tyrosine kinase inhibition of drug resistance pathway was observed to be associated with 20 DEGs, of which 17 were upregulated and three were downregulated. The EGFR is a tyrosine kinase that regulates cellular homeostasis, and gene mutations, as well as protein overexpression, activate downstream pathways and have been linked to a variety of malignancies (Huang and Fu, 2015). It was found here that the methylation level of EGRF in the blood of the control group was higher than that of the mastitis group. Additionally, the methylation ratios of EGFR genes were observed to increase in ovaries during the estrus phase compared with the diestrous phase ovaries (An et al., 2018). The hippo signaling pathway was discovered to be related to nine DEGs, five of which were upregulated and four downregulated. Hippo signaling is important in regulating organ size, tissue regeneration, and stem cell self-renewal (Zhao et al., 2011). The pathway involving inflammation regulation of the TRP channel was linked to 26 DEGs, 25 of which were upregulated and 1 downregulated. The *PLCG2*, *PIK3R2*, and *JAK2* genes were significantly enriched in this pathway; these genes were located at the core of the PPI network. Methylation of the PLCG2 promoter region was observed to be related to the regulation of immune activation during wildness training of giant pandas (Jie et al., 2022). *PIK3R2* gene is documented to promote the progression of malignancy through the PI3K/Akt/NF-κB axis, while METTL3 in glutamic acid-induced ICCs significantly accelerated the maturation of pri-miR-30b-5p by m6A methylation modification, reducing the levels of PIK3R2 and leading to the inhibition of the PI3K/Akt/mTOR pathway (Gong et al., 2022). The CpG islands in the promoter regions of *JAK2* and *STAT5A* gene were found to be hypomethylated, resulting in increased gene expression in cows with mastitis compared to healthy controls; the opposite was seen with the *CD4* gene (Usman et al., 2022). A SNP in the *JAK2* gene was observed to affect SCC values significantly, with significant dominant effects observed in the fat percentage (*p* < 0.05), suggesting that these could be used as potential epigenetic markers for the prediction of mastitis susceptibility in dairy cattle.

The distribution of DMRs over chromosomal regions revealed that the reads of the two groups of samples showed greater enrichment in the tail ends of the chromosomal regions relative to the other regions. The chromosomal terminus is a specific structure that consists of telomere and sub-telomere regions. In normal somatic cells, telomere shortening shortens human lifespans and reduces the incidence of age-related diseases. The levels of telomere methylation increase gradually with age, which is associated with telomere length reduction and cellular aging. In addition, changes in telomere methylation levels have been associated with a variety of health indicators and disease risks, such as cardiovascular disease, diabetes, and Alzheimer’s disease. The DNA repeat sequences in the telomere region are not methylated, whereas the sub-telomere region is enriched with a large number of CGIs and histone markers and is highly methylated. In most tumor cells, abnormally elevated levels of telomerase can maintain the telomere, allowing indefinite differentiation (Lu et al., 2019). In the present study, greater methylation was seen in the chromosomal termini in Xinjiang brown cattle, suggesting that the mastitis resistance of Xinjiang brown cattle may be related to high methylation levels of the chromosome terminus.

The *TRAPPC9* gene associated with various diseases, including intellectual developmental disorder, autosomal recessive disorder 13, and intellectual disability-obesity-brain malformations-facial dysmorphism syndrome. It is associated with pathways such as transport to the Golgi and vesicle-mediated transport. TRAPPC9 protein activates NF-κB by increasing phosphorylation of the IKK complex. Infection of the cow mammary gland by pathogenic microbes causes an increase in leukocyte numbers in the breast, as well as the release of huge amounts of inflammatory cytokines, which cause neutrophil accumulation and activation.Blood samples of Xinjiang brown cattle with different SCCs were collected to plot the methylation patterns of CpG sites; BSP assays revealed that the DMRs of the *TRAPPC9* gene were highly methylated. A comparison of the two groups revealed that *TRAPPC9* methylation was higher in the mastitis group than in the control group, while *TRAPPC9* gene expression was significantly lower in the low-SCC group (the control group) than in the high-SCC group (the mastitis group), indicating an opposite trend. Khan et al. found that the homozygous GG genotype in SNP3 of the *TRAPPC9* gene associated with high levels of IL-6 and low levels of SCC (Khan et al., 2022). Quantitative results revealed that GG-type individuals (low SCC) had much lower expression than TG-type persons (high SCC). In contrast to the trend of the observed expression levels, it remains to be investigated whether clinical mastitis is responsible for the change in the expression of this gene. CD4 molecules are mainly expressed on the surfaces of the thymus and mature T cells, where they play an important role in host resistance to viral and bacterial infections. Wang et al. reported that cows with CM had 16% more methyl groups in the bovine *CD4* promoter (75.0% ± 5.8%) than controls (59.0% ± 8.5%) (Wang et al., 2013), based on ATAC-seq maps of human CD4^+^ T cells acquired on consecutive days (Buenrostro et al., 2013). The reduced expression of the *CD4* gene in CM cows may be downregulated by the increased levels of methylation in the gene’s promoter region (Wang et al., 2013). The analysis of DMR methylation patterns in *CD4* gene in Xinjiang brown cattle revealed that methylation levels of both CpG2 and CpG4 in the control group were higher than those in the mastitis group, and the expression of *CD4* gene in the control group (low SCC) was significantly higher than that in the mastitis group (high SCC) (*p* < 0.05). The expression of this gene was negatively correlated with high methylation of *CD4* in the mastitis group. The DNA methylation map of Xinjiang brown cattle with different SCCs was analyzed at the genome-wide level, resulting in the identification of numerous differentially methylated genes, which were found to be mainly associated with disease resistance, growth, and metabolism. Future research will focus on elucidating the functions and regulatory mechanisms of mastitis resistance genes in Xinjiang brown cattle, as well as constructing key regulatory networks.

## 5 Conclusion

This study analyzed the genes and regulatory mechanisms associated with mastitis in Xinjiang brown cattle at the epigenetic level, identifying 1,934 DEGs potentially involved in the occurrence and development of mastitis. It was found that the expression of *TRAPPC9* and *CD4* genes was reduced in cows with mastitis, with the expression of *TRAPPC9* induced by inflammation showing the same trend. It is speculated that there might be a negative regulatory relationship between the expression of these genes and the occurrence of mastitis, the specific mechanism of which requires further investigation.

## Data Availability

The raw data supporting the conclusions of this article will be made available by the authors, without undue reservation.
