# Supplementary material for: DNA methylation patterns in the peripheral blood of Xinjiang brown cattle with variable somatic cell counts
Source: Front Genet. 2024 Jul 9;15:1405478. doi: 10.3389/fgene.2024.1405478 (PMC11263093; doi:10.3389/fgene.2024.1405478)
Supplement: Supplementary file 1 [file Table1.DOCX]

Supplementary Material

DNA methylation patterns in the peripheral blood of Xinjiang Brown Cattle with variable Somatic Cell Counts

Dan Wang^1^, Shengchao Ma^1^, Mengjie Yan^1^, Mingming Dong ^1^, Menghua Zhang^1^, Tao Zhang^1^, Tao Zhang^1^, Xiaoxue Zhang^1^, Lei Xu^1^, Xixia Huang^1*^

*** Correspondence:** Corresponding Author: au-huangxixia@163.com (X.H.)

# Supplementary Figures and Tables

## Supplementary Figures


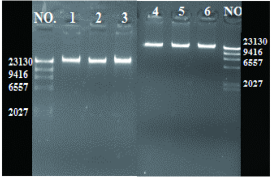


**Supplementary Figure 1.** The agarose gels electrophoretic patterns of bovine genomic DNA.


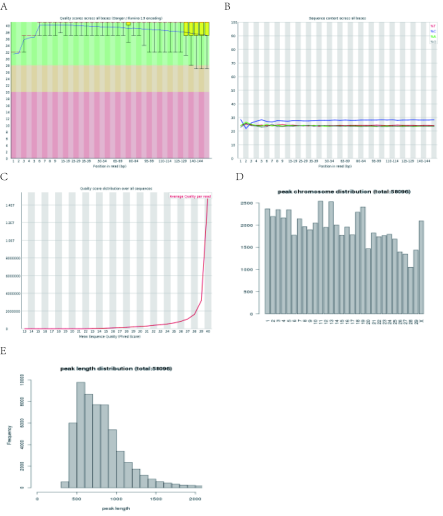


**Supplementary Figure 2.** MeDIP-Seq data quality assessment. (A) Q value box statistics. (B) base distribution (C)base quality (D)Distribution of *peak*s on Chromosome (E) *Peak* length distribution.


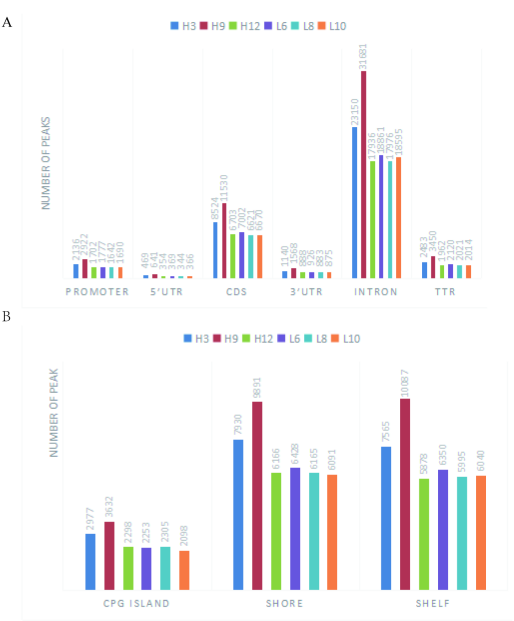


**Supplementary Figure 2.** MeDIP-Seq data quality assessment. (A) Q value box statistic (B) base distribution (C) base quality (D) Distribution of *peak*s on Chromosome (E) *Peak* length distribution.


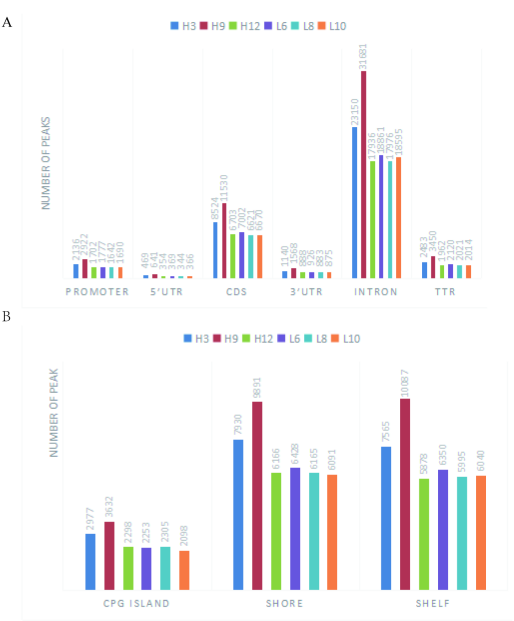


**Supplementary Figure 3.** *Peaks* on different genetic elements.


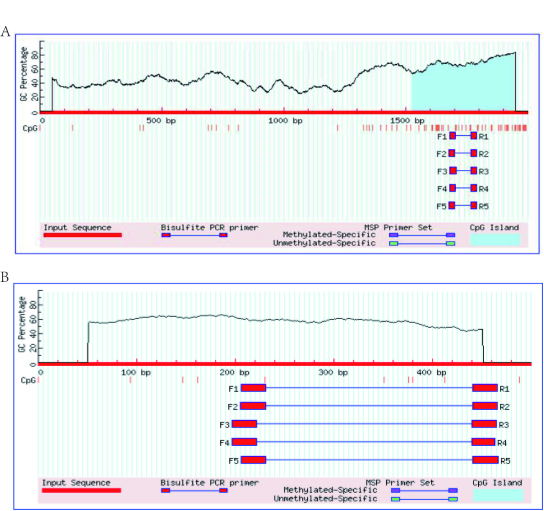


**Supplementary Figure 4.** The *CpG* prediction of TRAPPC9 and CD4.


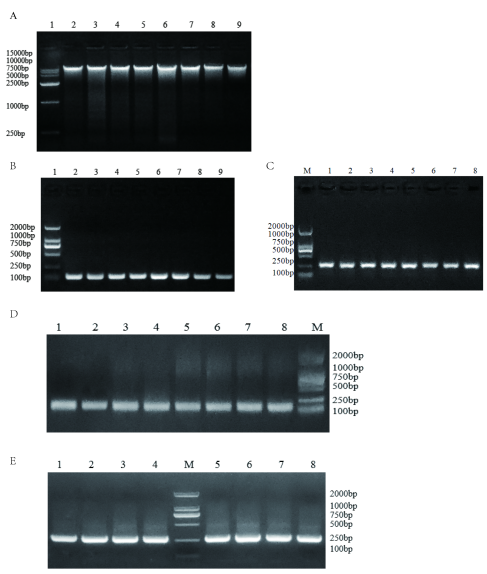


**Supplementary Figure 5.** DNA and PCR product detection. (A) Gel electrophoresis of genomic DNA of Xinjiang brown cattle (B) PCR product of TRAPPC9 gene (C) PCR product of CD4 gene (D) TRAPPC9 gene bacteriophage PCR product (E) CD4 gene PCR bacteriophage PCR product.

M is DL2000 DNA Marker; 2-4 are 4 Xinjiang brown cows with clinical mastitis; 5-9 are 4 Xinjiang brown cows with normal lactatio
